# Symptom appraisal and help seeking in males with symptoms of possible prostate cancer: a qualitative study with an ethnically diverse sample in London

**DOI:** 10.3399/BJGP.2022.0554

**Published:** 2023-05-31

**Authors:** Ben Shaw, Fiona M Walter, William Hamilton, Tanimola Martins

**Affiliations:** College of Medicine and Health, University of Exeter, Exeter.; Wolfson Institute of Population Health, Barts and The London School of Medicine and Dentistry, Queen Mary University of London, London; Primary Care Unit, Department of Public Health and Primary Care, University of Cambridge, Cambridge.; College of Medicine and Health, University of Exeter, Exeter.; College of Medicine and Health, University of Exeter, Exeter.

**Keywords:** ethnicity, help seeking, men, primary care, prostate cancer, symptoms

## Abstract

**Background:**

Prostate cancer mortality in Black males is disproportionately high. This problem may be overcome by reducing delays in the pathway to diagnosis, particularly those occurring before initial medical help seeking. A greater understanding of symptom appraisal and help seeking could support the development of targeted interventions for improving early presentation among Black males.

**Aim:**

To provide an in-depth understanding of males' pre-consultation experiences following the onset of symptoms of possible prostate cancer, identifying both general trends as well as potential differences that may exist between Black and White males.

**Design and setting:**

Qualitative study of 18 males (nine Black, nine White) in London, UK, who had recently seen their GP with urinary symptoms, erectile dysfunction, or haematuria.

**Method:**

Semi-structured interviews from a previous multi-methods study of primary care use by males with symptoms of possible prostate cancer were analysed using thematic framework analysis.

**Results:**

Symptoms were often interpreted by patients as unimportant. Most delays occurred due to the absence of reasons to seek help, which, in Black males, often stemmed from poor awareness of prostate cancer. This lack of awareness could have been a consequence of their reluctance to seek health information and discuss health issues with others in their social network. Friends and relatives played an important role in symptom appraisal and help seeking.

**Conclusion:**

Cognitive biases, cultural stigmas, and everyday interpersonal interactions should be important areas at which to target strategies seeking to reduce delays and improve early presentation in males with possible prostate cancer, particularly Black males.

## INTRODUCTION

Prostate cancer is the most common cancer among UK males and a leading cause of cancer deaths each year.^1^ There are important differences in prostate cancer incidence and mortality by ethnicity in the UK:^1^ the incidence is about three times higher in Black males, compared with White males, in part due to biological factors resulting from complex gene–environment interactions.^2^ The case-specific mortality rate is 35% greater in Black males compared with White males,^3^ which may be linked to a greater incidence of advanced-stage diagnoses in those of Black ethnicity.^4^ Although Black males more frequently develop aggressive subtypes of prostate cancer,^5^ tumour characteristics may not fully explain the differences in advanced-stage diagnoses and, by extension, the higher mortality among males in this broad ethnic group.^4^^,^^6^ Advanced-stage diagnoses may result from poor access to health care, lower socioeconomic status, and other structural factors,^7^ although there is currently no clear evidence to support this explanation in the context of free universal health care in the UK.^8^

Advanced-stage diagnosis may also arise from delays in the pathway to diagnosis.^9^ Despite doubt surrounding the link between lower urinary tract symptoms (LUTS) and prostate cancer,^10^^,^^11^ the absence of prostate cancer screening in the UK means that diagnosis most commonly occurs following presentation to primary care with LUTS, erectile dysfunction, or visible haematuria.^11^^,^^12^ However, a multi-methods study of UK males showed that Black males may not fully disclose possible prostate cancer symptoms during initial consultation in primary care; in addition, the study found that GPs may delay investigation of males in this group, especially if patients have comorbidities, such as diabetes, that share similar symptoms.^13^ These differences may lead to delays and a prolonged diagnostic interval (the period between initial primary care consultation and diagnosis).^14^^,^^15^ Delays may also arise in the time between symptom onset and presentation to health care (patient interval):^14^^,^^15^ it has been reported that Black males may misinterpret possible symptoms due to a lack of awareness of or knowledge about prostate cancer,^16^^,^^17^ and may delay help seeking due to complex psychosocial and structural barriers related to fear, stigma, fatalism, and religious/cultural beliefs and norms.^16^^,^^18^^,^^19^ Recent studies, however, found no statistical evidence of ethnic differences in patient-interval duration and only a small difference in diagnostic interval to explain the mortality gap in prostate cancer.^13^^,^^20^ As such, it has been assumed that inequalities in symptomatic diagnosis are unlikely to be a major cause of ethnic inequalities in prostate cancer outcomes among UK males.^21^ Yet, a significant proportion of males (including Black males) in the UK delay presenting by at least 12 months following the onset of symptoms,^13^ meaning there may still be scope to tackle the mortality gap by reducing delays prior to primary care consultation. Doing so necessitates an understanding of symptom appraisal and help-seeking processes preceding presentation.

**Table:** How this fits in

Most Black males with prostate cancer are diagnosed after presenting with symptoms to primary care, and — while unlikely to be the main explanation for the greater mortality in this group — an understanding of the events during this pathway could inform strategies to tackle inequalities in prostate cancer outcomes through earlier diagnosis. Existing evidence highlights the influence of symptom appraisal and help seeking in cancer diagnostics, although it is unclear how this applies to Black males in the UK with symptoms of possible prostate cancer. The present study gives clarity to this issue, highlighting the potential for improving early presentation in symptomatic Black males, while illustrating the role that GPs can play in facilitating early diagnosis.

Martins *et al*^13^ reported findings from quantitative data on ethnic differences in the patient interval, but no study has specifically explored males' pre-consultation experiences in the UK. This means that important ethnic differences may still exist in a qualitative analysis. A greater understanding of symptom appraisal and help seeking could provide insights that support the development of interventions targeted at improving early presentation among Black males. Likewise, any benefit gained from this understanding may also extend to other ethnic groups.

In the present study, the authors performed a secondary analysis of qualitative data from males who had recently reported symptoms of possible prostate cancer in UK primary care. They aimed to provide an in-depth understanding of males' pre-consultation experiences following the onset of possible prostate cancer symptoms, identifying both general trends as well as potential differences that may exist between Black and White males.

## METHOD

Qualitative data from a previous multi-methods study of primary care use by an ethnically diverse sample of males with symptoms of possible prostate cancer^13^ were thematically analysed. Sampling, recruitment, and data collection procedures are detailed elsewhere,^13^ but outlined briefly below.

### Setting and participants

Between March 2016 and October 2017, patients presenting to general practices with symptoms of possible prostate cancer were recruited in, and around, London, UK. Practices were recruited to the study with the help of the National Institute for Health and Care Research Clinical Research Network. The authors searched practices' electronic health records for patients aged ≥40 years, who attended the practice with LUTS, erectile dysfunction, and/or haematuria (with or without other non-specific cancer symptoms — for example, weight loss) in the previous 6 months. Those with benign prostatic hyperplasia or a previous history of prostate cancer were excluded.

Potential participants were surveyed via secured postal services and a subset was invited to participate in face-to-face interviews. Participants provided self-defined ethnicity, which was then merged into combined ethnic groups by the study team, based on the UK census definition. Interviewees were purposively sampled based on age, level of education, and ethnicity. Twenty-three males participated in the face-to-face interviews: nine were Black, nine were White, and five were Asian.

### Data collection and processing

Interviews were conducted at each participant's home or workplace, and lasted approximately 60 minutes. The interviews were semi-structured, using a schedule developed from similar interviews undertaken with people referred with possible cancer or recently diagnosed with cancer (Supplementary Information S1).^22^^,^^23^ The interviews explored males' experiences, from noticing symptoms of possible prostate cancer to being offered investigations. Recruitment ended when thematic saturation was reached — that is, when no new information pertinent to the study was identified.^24^

All interviews were audio-recorded, professionally transcribed verbatim, and anonymised via a confidential service. The interview data were coded and analysed by three researchers in the previous study, which focused mainly on the primary care phase of the patients' pathway. In the present study, a fourth researcher performed a secondary analysis of the data, focusing on the pre-presentation phase only, with support from one of the initial researchers.

### Data analysis

Thematic framework analysis was used with an iterative analytical process that followed five stages:

- familiarising oneself with the data;
- developing a thematic framework;
- applying data to the framework;
- mapping; and
- questioning the data and thematic interpretation.^25^

The conceptual framework was developed using an inductive–deductive approach, guided by the data and the Model of Pathways to Treatment framework.^14^ The thematic framework focused on the patient interval, with codes being grouped into four categories based on key events occurring prior to primary care presentation:

- initial appraisal of symptom(s);
- consequences of symptom(s);
- responses to symptom(s); and
- re-appraisal and help seeking.

New codes were identified and relevant codes from the original analysis were applied to this framework. Data were charted into a coding matrix to aid comparison across ethnic groups and facilitate the development of themes. A phenomenological approach was adopted and the authors searched for semantic-level themes, drawing on the most commonly discussed attitudes and experiences. The primary researcher was a clinician, whose experience in consulting with males presenting to primary care with cancer symptoms is reflected in the interpretation of themes.

## RESULTS

The study comprised 18 males (Black, *n* = 9; White, *n* = 9). The Asian males, who had been interviewed in the initial study, were not included as insufficient data relating to the pre-presentation phase in that group greatly limited interpretation of the findings. Participants were aged 44–75 years and had varied educational backgrounds and employment statuses. A detailed breakdown of participant characteristics is shown in Table 1.

**Table 1.** Participants' characteristics by ethnicity

**Characteristic**	**Interview participants, *n* (%)^a^**		

	**Total**	**White**	**Black**
**Total**	18	9	9

**Age, years, median (IQR)**	62 (57–70)	64 (58–70)	60 (57–68)

**Employment status**			
Employed	7 (38.9)	2 (22.2)	5 (55.6)
Unemployed	2 (11.1)	2 (22.2)	0 (0.0)
Retired	9 (50.0)	5 (55.6)	4 (44.4)
Sick/disabled	0 (0.0)	0 (0.0)	0 (0.0)
Other	0 (0.0)	0 (0.0)	0 (0.0)

**Highest educational qualification**			
Degree/diploma/equivalent	11 (61.1)	6 (66.7)	5 (55.6)
A-level/GSCE/O level	6 (33.3)	2 (22.2)	4 (44.4)
Other/none	1 (5.6)	1 (11.1)	0 (0.0)

**Smoking status**			
Current smoker	1 (5.6)	1 (11.1)	0 (0.0)
Ex-smoker	5 (27.8)	4 (44.4)	1 (11.1)
Never smoked	12 (66.7)	4 (44.4)	8 (88.9)

**Alcohol intake**			
Current drinker of alcohol	10 (55.6)	9 (100.0)	1 (11.1)
Ex-drinker of alcohol	5 (27.8)	0 (0.0)	5 (55.6)
Never drank alcohol	3 (16.7)	0 (0.0)	3 (33.3)

**Comorbidity** ^b^	10 (55.6)	4 (44.4)	6 (66.7)

**Family history**			
Diabetes	6 (33.3)	4 (44.4)	2 (22.2)
Cancer	5 (27.8)	3 (33.3)	2 (22.2)
Heart disease	8 (44.4)	5 (55.6)	3 (33.3)

**Live alone**	7 (38.9)	3 (33.3)	4 (44.4)

a *Unless specified otherwise.*

b *Comorbidity includes diabetes, kidney disease, bladder disease, incontinence, Parkinson's disease, brain and spinal cord disease, anxiety and depression, and other cancers. GCSE = General Certificate of Secondary Education. IQR = interquartile range.*

The most commonly experienced symptoms were increased urinary frequency/nocturia (*n* = 11), urgency/urge incontinence (*n* = 10), and erectile dysfunction (*n* = 9). One-third of males initially saw their GP for a reason other than their symptoms of possible prostate cancer (*n* = 6) and, in those whose symptoms were the reason for attending, erectile dysfunction was rarely the primary reason (*n* = 2). Five males had attended more than once for the same symptom(s) without previous investigation. Almost all males with LUTS or erectile dysfunction waited at least 3 months following the onset of symptoms to seek help; five males waited >1 year and two waited almost 10 years. Males with haematuria, however, presented within days or weeks after first noticing the symptom. The most significant delays arose from males not perceiving a reason to seek help because their symptoms were stable, attributable, manageable, and/or not severe enough to greatly affect their life (data not shown).

Findings are presented, grouped by subthemes under four headings; each heading relates to a category used in the thematic coding and a key stage of the pre-presentation pathway. The interdependence between each process means there is marked overlap between the timing and content of each subtheme. Each subtheme is outlined using illustrative quotations to explore how biological, psychosocial, and structural factors influenced symptom appraisal and help seeking.

### Initial appraisal of symptom(s)

#### Symptom attribution

Males were keen to understand the cause of their symptoms and often adopted an analytical approach:

*‘I don't drink, I don't smoke, I don't do drugs … Why would I have a problem like this?’*

(B3, Black, aged 50–59 years)

Most attributed their symptoms to getting old, medication, or their eating or drinking habits:

*‘I'm a pub goer. I drink beer and it tends to be later in the evening, so I'm going up to go to the loo … in the night, anyway.’*

(W1, White, aged 70–79 years)

Many Black males who had diabetes attributed their symptoms to that, based on their GP's advice:

*‘The doctor said “go and shed weight” … He didn't ask me too much questions, because diabetes has explained everything to him.’*

(B8, Black, aged 60–69 years)

Erectile dysfunction was rarely seen to be linked to urinary symptoms or possible prostate pathology, except in relation to diabetes:

*‘I was thinking is it because I … I don't have enough sex with my wife, or is it because I sit on the scrotum most of the time?’*

(B6, Black, aged 60–69 years)

#### Symptom knowledge and recognition

Some males believed that they could not have a prostate issue because they were not old enough. Only three males, all of whom were White, recognised that their symptoms could indicate prostate cancer. These males knew family and friends with prostate issues:

*‘Immediately, I thought, I've got a prostate problem … it seems to be the thing to have at the moment … so many of my friends are suffering from prostate issues.’*

(W6, White, aged 60–69 years)

*‘Everyone you talk to in the allotments … they're all having problems.’*

(W8, White, aged 70–79 years)

However, most males felt that their symptoms would settle down by themselves or could not be treated:

*‘I don't know why you put it off … it's more about thinking, “Well, what can they do?”, yeah?’*

(W4, White, aged 50–59 years)

*‘I don't think there's anything the doctors could do that would help.’*

(B2, Black, aged 70–79 years)

### Consequences of symptom(s)

#### Emotional impact

Both Black and White males were embarrassed by their symptoms, particularly the thought of wetting themselves:

*‘You run into a bush or somewhere to start weeing against somebody's wall or somebody's garden … If I don't wee at that time, I'm gonna be wet.’*

(B3, Black, aged 50–59 years)

*‘I wouldn't completely urinate before I reached the lavatory, but I would dribble in a way which I found very unpleasant and unattractive.’*

(W7, White, aged 70–79 years)

Black males often felt unable to talk to friends about their symptoms because they were embarrassed about being labelled as an old man. Some males wanted *‘a quick answer, like, you know, a reassurance’* (B2, Black, aged 70–79 years), while others *‘didn't lose any sleep thinking about it’* (W9, White, aged 60–69 years) because they could rationalise their symptoms. White males were generally more fearful about the potential cause of their symptoms, often because they had haematuria, which they linked to something serious:

W3 (White, aged 50–59 years): *‘There is an initial, kind of, panic …’*

Interviewer: *‘When you first saw the blood?’*

W3: *‘Yeah.’*

#### Quality of life

Urinary symptoms greatly affected quality of life:

*‘You don't sleep … I seriously think I urinated five times.’*

(W4, White, aged 50–59 years)

*‘Let's say my girlfriend is lying next to me … I have to jump over her to go and wee three or four, five times … It makes* [her] *mad.’*

(B3, Black, aged 50–59 years)

They were also a particular issue in males who did not have ready access to a toilet due to their job:

*‘As a part-time Uber driver, I need my drink while driving but passenger in your car, no time for a wee … by the time I get there, I've wee'd myself.’*

(B3, Black, aged 50–59 years)

*‘I'd think of which stations had got a good toilet … it was costing me a fortune getting on and off the tube* [to attend business meetings].’

(W9, White, aged 60–69 years)

### Responses to symptom(s)

#### Coping and self-management

Most males initially self-managed their symptoms because they felt they were inconvenient but relatively manageable:

*‘I just come in and strip off and wash my clothes.’*

(W9, White, aged 60–69 years)

White males often modified their lifestyle by drinking less tea and coffee in the evenings. Black males commonly experimented with home remedies but reported poor success:

*‘We're from Barbados, and they have all kind of remedies over there … There was some slimy thing … it was horrible but I forced it down. Waste of bloody time.’*

(B4, Black, aged 60–69 years)

Many males adapted their lives to manage the consequences of their symptoms:

*‘I walk with a spare pair of trousers, just in case.’*

(B2, Black, aged 70–79 years)

#### Seeking information

White males openly discussed their problems and would *‘joke about men of a certain age’* (W4, White, aged 50–59 years) with their friends, whereas Black males were more likely to see it as a taboo subject:

*‘If I met you as a man, and we're mates, I'm not gonna come and tell you and say, “Oh, guess what?”’*

(B5, Black, aged 50–59 years)

*‘It's a little bit more personal, isn't it? … Other people don't talk about their personal things.’*

(B9, Black, aged 60–69 years)

Many males discussed reservations about using the internet to search for information about their symptoms, although several males (mostly White) still did:

*‘I looked at it, Google, but once you open Google you can find, you know, one million things … most of them quite extreme.’*

(W2, White, aged 50–59 years)

Black males were less likely to use the internet:

*‘I wouldn't know where to start … I get frustrated.’*

(B4, Black, aged 60–69 years)

They would often not seek any information at all and, when they did, they sometimes obtained misinformation:

*‘Back home when you have a weak erection … they call it jedijedi* [haemorrhoids]*, isn't it?’*

(B6, Black, aged 60–69 years)

*‘From what I've heard … it* [Viagra] *gives you heart failure.’*

(B5, Black, aged 50–59 years)

### Re-appraisal and help seeking

#### Deciding to seek help

Males would decide to actively seek help once they perceived their symptom(s) to be important — and, so, had a reason to seek help — and were then triggered by a specific stimulus. Both were achieved when symptoms had not improved, or when they had increased in frequency/severity over a short period of time and became severely detrimental to quality of life. Many males perceived symptoms to be important once attempts to self-manage them became ineffective, because that inefficacy caused them to question their initial symptom attribution:

*‘I couldn't rationalise it … I couldn't put a definite finger on it.’*

(B1, Black, aged 50–59 years)

The nature of the symptom(s) was also important, with haematuria frequently causing immediate concern as it was associated with a serious cause:

*‘If you've got blood in your urine, something's wrong, obviously.’*

(W5, White, aged 40–49 years)

Symptoms that were considered to be abnormal in males' social networks were also important:

*‘I have a couple of friends in my age group … who are still very active sexually and I think, “Oh, right, well, what's wrong with me?”’*

(B4, Black, aged 60–69 years)

Once males perceived a reason to seek help, it was often encouragement from their partner or friends that triggered them to book an appointment:

‘[My wife] *just kept on at me about the colour of my urine*.’

(B4, Black, aged 60–69 years)

*‘My friend was checking me: “Have you had chance? Have you checked with the docs yet?”’*

(B2, Black, aged 70–79 years)

White males, in particular, were triggered by gaining an awareness about the dangers of prostate cancer from friends:

*‘A number of friends of mine have suffered from prostate cancer … it's obviously so common in men that I thought, well, perhaps I ought to go.’*

(W1, White, aged 70–79 years)

#### Accessing help

Some males struggled to take time off work; others worried about wasting their GP's time:

*‘You don't want to make a big drama of little things in case you call the doctor and it turns out to be nothing.’*

(B9, Black, aged 60–69 years)

Black males often had multiple symptoms that they did not have time to disclose during the consultation:

*‘You have limited time … if you accumulate complaints, you won't be able to discuss* [them].’

(B8, Black, aged 60–69 years)

Males also often struggled to access their GP due to an absence of walk-in appointments outside of normal working hours. Several males were put off because they were unsure whether they would be taken seriously by their GP if their symptoms could be attributed to a non-serious cause. Anxiety and embarrassment about seeing the GP was also common among Black and White males because it was perceived to threaten their masculinity:

*‘We men … we don't want to own up that we're not good.’*

(B5, Black, aged 50–59 years)

*‘I was very … very self-conscious and embarrassed at the prospect of a rectal examination.’*

(W7, White, aged 70–79 years)

Despite embarrassment about the prospect of a digital rectal examination (DRE), most males were not deterred from seeing their GP and were happy to accept investigations if it could help to uncover the cause of their symptoms. The GP's sex was an important factor in help seeking, however, and some males said they would have been discouraged from disclosing their symptoms if the GP was not of their preferred sex:

Interviewer: *‘If it was a female doctor, would this have affected …?’*

W8 (White, aged 70–79 years): *‘Yeah, I probably wouldn't have told her* [about urinary symptoms] *… I'd have been too embarrassed.’*

However, no clear overall sex preferences or patterns emerged within, or across, ethnic groups; some males preferred to see a male GP, some preferred to see a female GP, and others were indifferent to the consulting GP's sex.

## DISCUSSION

### Summary

Symptoms were interpreted in the context of existing lifestyle choices and comorbidities, and were often attributed to these factors. Symptoms that negatively affected quality of life were often stable and manageable, and, as such, males did not initially perceive them to be important. Participants perceived a reason to seek help following changes in symptom nature, duration, frequency, severity, and their impact on daily life, or once they could not explain the cause of their symptoms after attempts to self-manage became ineffective. Black males were less likely to recognise that their symptoms could indicate prostate cancer, possibly as a consequence of their reluctance to seek health information or discuss health issues with others in their social network. Friends and relatives influenced symptom appraisal and often triggered the decision to seek help, which may link with these differences. Most men encountered personal or emotional barriers to help seeking, including time and embarrassment, although this did not appear to cause marked delays to presentation in either group.

### Strengths and limitations

To the authors' knowledge, this is the first study to directly explore ethnic differences in symptom appraisal and help seeking in males with symptoms of possible prostate cancer. The age range and demographic diversity of the sample provided a wealth of material with which to identify general trends and highlight specific differences between Black and White males. In addition, qualitative themes were structured using the Model of Pathways to Treatment framework, thereby facilitating comparison with any existing or future studies. Likewise, the findings may be transferable to other urological pathologies, given the general nature of symptoms explored.

As the analysis in the present study was restricted to symptom appraisal and help seeking in Black and White males, the findings may not apply to other stages of cancer diagnostic pathways or males of other ethnicities. In addition, discussions about symptom appraisal and help seeking typically focused on events that had started several years before primary care consultation and participation in the interviews; as such, a degree of recall bias is possible. Furthermore, as data for the present study predate the COVID-19 pandemic, the findings may not fully reflect males' experiences during this period or how the pandemic may have changed symptom appraisal and help-seeking behaviour.

Sociodemographic factors (such as age, comorbidity, education, and employment status) were used primarily for identification purposes. These factors may influence symptom appraisal/help seeking^26^ and prostate cancer survival,^27^ but how they interacted with ethnicity to produce the accounts given by participants was not explored. Although this is beyond the scope of this study, it is important to recognise that differences in these individual characteristics between the Black and White ethnic groups may have influenced the findings; as an example, comorbidity rates were higher in the group of Black males and, due to symptom misattribution, this may have contributed to their lack of recognition of symptoms.

### Comparison with existing literature

Previous studies have highlighted the importance of cognitive heuristics in symptom appraisal, most notably the rate-of-change rule and the severity (of interference) rule^28^ — that is, symptoms that are either quickly worsening or negatively affecting daily life are more likely to be interpreted as indicating illness. These ‘rules of thumb’ may be normalised when they are common in a social network,^26^ and the results presented here support this; this is demonstrated by the significant delays that occurred in the absence of these factors, which was a common finding in those with urinary symptoms.^29^ This suggests that strategies that seek to improve early presentation in Black males should tackle symptom misinterpretation by focusing on cognitive biases, rather than specific symptom awareness, as posited by Kummer *et al*.^28^ In addition, it emphasises the importance of considering facilitators to help seeking in the Model of Pathways to Treatment framework, while demonstrating the relevance of the psychological theory by which it is underpinned.

Research on symptom appraisal and help seeking in minority ethnic groups has classically focused on barriers to seeking help, such as masculinity, fear, and poor access to health care.^16^^,^^19^^,^^30^^,^^31^ Although evident in the data presented here, these were not specific to Black males and did not appear to account for significant delays. More apparent was that there was an absence of reasons to seek help, which often stemmed from a poor awareness, in Black males, of prostate cancer — a common finding in minority ethnic groups with possible cancer symptoms.^16^^,^^18^ Black males' attitudes towards seeking health information and discussing health with close contacts may be related to cultural stigmas that undermine the legitimacy of discussing health.^22^^,^^31^^–^^33^ This may result in fewer proximal social cues (that is, everyday interpersonal interactions with friends, family, and colleagues) that help males to either recognise and understand the importance of their symptoms, or to seek help as a result of their concerns being legitimised and additional information being provided.^17^^,^^29^^,^^32^^,^^34^^,^^35^ Overcoming cultural stigmas associated with prostate cancer and providing proximal social cues should, therefore, be a focus of future strategies for improving early presentation in Black males.

The work presented here offers a possible explanation for why no difference has been found in the duration of the patient interval between Black and White males, despite notable differences in their symptom appraisal and help-seeking behaviours: although Black males are, typically, less aware about the risks of prostate cancer than White males, they may also be less successful at managing their own symptoms and, as such, perceive a reason to seek help sooner than they otherwise would. As a result, while inequalities in symptomatic presentation and diagnosis may not be a major cause of ethnic inequalities in prostate cancer outcomes in the UK, this study highlights the potential that remains for targeted interventions to improve early presentation in symptomatic Black males.

### Implications for practice

Future research should continue to build on recent work that has applied knowledge of symptom appraisal and help seeking to develop interventions that promote early presentation in Black males — and, in particular, strategies targeting symptom misinterpretation, cultural stigmas, and proximal social cues.^36^^–^^38^ The finding that symptoms were easily attributed to other causes, and rarely perceived as a problem, has several important implications for clinical practice because Black males may not present with their symptoms until they reach an advanced stage of disease, if at all. This means that GPs should actively ask about symptoms and, in order to avoid further delays, have a low threshold for prostate-specific antigen (PSA) testing and 2-week-wait referral in Black males when there are doubts surrounding DRE findings. Whether GPs should actively offer a PSA test to Black males aged >50 years without them having to request it first (as is stipulated by current guidance from the National Institute for Health and Care Excellence)^39^ could also be considered. This reflects a potentially necessary shift in focus towards asymptomatic diagnosis, especially given ongoing uncertainty about the value of urinary symptoms in early-stage detection of prostate cancer.^11^ This could also support the case for targeted prostate cancer screening to identify both those who are asymptomatic and those who have symptoms but do not present or disclose their symptoms in primary care.
